# Supplementary material for: The photo-inhibition of camphor leaves (Cinnamomum camphora L.) by NaCl stress based on physiological, chloroplast structure and comparative proteomic analysis
Source: PeerJ. 2020 Aug 7;8:e9443. doi: 10.7717/peerj.9443 (PMC7486828; doi:10.7717/peerj.9443)
Supplement: Supplemental Information 1 [file peerj-08-9443-s001.docx]

Abbreviation

| Chl | Total chlorophyll |
| --- | --- |
| Chl a | Chlorophyll a |
| Chl b | Chlorophyll b |
| Car | Total carotenoids |
| SOD | Superoxide dismutase |
| POD | Peroxidase |
| MDA | Malondialdehyde |
| *A*_n_ | Photosynthetic rate |
| *g_s_* | Stomatal conductance |
| *C*_i_ | Intercellular CO_2_ concentration |
| *WUE_i_* | The intrinsic water use efficiency |
| *C*_i_/*C*_a_ | The ratio of intercellular to ambient CO_2_ concentration |
| F_o_ | Minimal chlorophyll fluorescence emission |
| F_m_ | Maximal chlorophyll fluorescence emission |
| F_o_' | Minimal fluorescence level in the light-adapted state |
| F_m_' | Maximal fluorescence level in the light-adapted state |
| F_s_ | The steady-state value of fluorescence |
| qp | Photochemical quenching coefficient |
| F_v_/F_m_ | Maximum quantum yield PSII |
| H_d_ | Thermal dissipation |
| NPQ | Non-photochemical quenching |
| PM | plasma membrane |
| CW | cell wall |
| ChM | chloroplast membrane |
| SG | starch granules |
| OS | osmium granules |
| Thl | lamellar structure of thylakoids |
| iTRAQ | Isobaric tags for relative and absolute quantitation |
| PSI | Photosystem I |
| PSII | Photosystem II |
| LHC | Light-harvesting complex |
| DEPs | Differentially expressed proteins |
